# Mental health policy in Eastern Europe: a comparative analysis of seven mental health systems

**DOI:** 10.1186/1472-6963-14-42

**Published:** 2014-01-27

**Authors:** Martin Dlouhy

**Affiliations:** 1 School of Informatics and Statistics, University of Economics in Prague, 4 Winston Churchill Square, 13067 Prague, Czech Republic

**Keywords:** Mental health policy, Health financing, Eastern Europe

## Abstract

**Background:**

The objective of this international comparative study is to describe and compare the mental health policies in seven countries of Eastern Europe that share their common communist history: Bulgaria, the Czech Republic, Hungary, Moldova, Poland, Romania, and Slovakia.

**Methods:**

The health policy questionnaire was developed and the country-specific information was gathered by local experts. The questionnaire includes both qualitative and quantitative information on various aspects of mental health policy: (1) basic country information (demography, health, and economic indicators), (2) health care financing, (3) mental health services (capacities and utilisation, ownership), (4) health service purchasing (purchasing organisations, contracting, reimbursement of services), and (5) mental health policy (policy documents, legislation, civic society).

**Results:**

The social and economic transition in the 1990s initiated the process of new mental health policy formulation, adoption of mental health legislation stressing human rights of patients, and a strong call for a pragmatic balance of community and hospital services. In contrast to the development in the Western Europe, the civic society was suppressed and NGOs and similar organizations were practically non-existent or under governmental control. Mental health services are financed from the public health insurance as any other health services. There is no separate budget for mental health. We can observe that the know-how about modern mental health care and about direction of needed reforms is available in documents, policies and programmes. However, this does not mean real implementation.

**Conclusions:**

The burden of totalitarian history still influences many areas of social and economic life, which also has to be taken into account in mental health policy. We may observe that after twenty years of health reforms and reforms of health reforms, the transition of the mental health systems still continues. In spite of many reform efforts in the past, a balance of community and hospital mental health services has not been achieved in this part of the world yet.

## Background

If something is true about mental illness then it is underestimation of its impact by both the governments and the public. According to the World Health Organisation, mental illnesses affect more than 25% of all people at some time during their lives. Mental illnesses are universal, affecting people of all countries and societies, individuals at all ages, women and men, the rich and the poor, from urban and rural environments. Mental illnesses have an economic impact on societies and on the quality of life of individuals and families. It has been estimated that as many as 450 million people suffer from mental illnesses and that four of the six leading causes of years lived with disability are due to mental illness. The global burden of neuropsychiatric disorders measured in disability-adjusted life years accounted for 13.1% of the total global burden of disease in 2004 [1]. It is evident that governments have to allocate adequate financial, material and human resources to address the health problem of this scale.

The objective of this study is to describe and compare the mental health policies and mental health systems in seven countries of Eastern Europe: Bulgaria, the Czech Republic, Hungary, Moldova, Poland, Romania, and Slovakia. Since the fall of communist regimes in 1989, Eastern European health systems (mental health systems included), went through many changes: health care financing out of taxation was replaced by public health insurance; former hierarchical state structures of health care delivery were replaced by health insurance agencies and independent public or private health care providers; many patient-oriented advocacy groups were formed; human rights of the mentally ill became an important issue.

According to performance of the national economy (Table 1), the economies of the Czech Republic, Hungary, Poland, and Slovakia are relatively stronger than the economies of three southern countries (Bulgaria, Moldova, and Romania). Economic performance of Moldova is very low in comparison to other European countries. All seven national economies perform below the European Union average (Table 1). From the year 2009, we can observe the impact of global economic crisis and consequently signs of mild recovery in the following years. In spite of unfavourable initial social and economic conditions, six countries, with the exception of Moldova, became members of the European Union.

**Table 1:** Gross domestic product in purchasing power parity (US dollar) per capita, 2011

**Country**	**GDP**
Bulgaria	14603
Czech Republic	25949
Hungary	21738
Moldova	3391
Poland	21281
Romania	15163
Slovakia	24433
European Union	32860

Source: European Health for All Database.

## Methods

Mental health system can be defined as the structure of institutions and all activities whose primary purpose is to promote, maintain or restore mental health. It does not include institutions or resources outside the health system, although we know that the care about mentally-ill needs a comprehensive system of services and coordination of health and social services is essential in case of mental health care. This is likely the main limit of the study. In order to describe and compare mental health systems, various methodologies can be used. We will mention two methodologies with which there is an international experience available. One successful approach to analyzing national health systems was developed by the *European Observatory on Health Systems and Policies*. The European Observatory produces *HiT Health Systems Reviews* (HiTs), which are country-based reports that provide a detailed description of a health system and reform and policy initiatives in progress. HiTs are prepared according to a template that is revised periodically and provides detailed guidelines, definitions, suggestion for data sources, and examples. Although the template is rather comprehensive and detailed, it is intended to be used in a flexible way to allow authors to adapt to their particular national context. An example of approach designed specifically for mental health was developed by the World Health Organisation. The *WHO Assessment Instrument for Mental Health Systems* (WHO-AIMS) was developed as a tool for comprehensive assessment of national mental health systems by a quite large set of input and process indicators. The idea behind the WHO-AIMS is that essential information needed for planning in order to strengthen mental health systems many countries has been lacking. In this study, we were inspired by both methodologies mentioned above. We made decision to adhere more to the HiTs methodology for the reason that we are interested not only in numbers, but particularly in the context and the story behind them.

During the project, the health policy questionnaire was developed [2] and the country-specific information was gathered by local experts (health policy analysts from universities and research institutions). The questionnaire includes both qualitative and quantitative information and covers various aspects of mental health policy in five sections: (1) basic country information (demography, health, economic indicators), (2) health care financing (public and private sources of financing), (3) mental health services (capacities and utilisation, ownership), (4) health service purchasing (purchasing organisations, contracting, reimbursement of services), and (5) mental health policy (existing policy documents, legislation, the roles of research and civic society). The information for fulfilling the questionnaire for was gathered by country experts in the years 2010–2012. The information collected in the questionnaires comes from the national health statistics, national health legislation, strategic health policy documents and communications with local stakeholders. The availability and reliability of the data on mental health services highly differs among the countries.

## Results

### Health care financing

In all the seven countries, the tax-based financing system was transformed into a public health insurance system. The transformation of health care financing was carried out during the 1990s with the exception of Moldova, which introduced a public health insurance only in 2004. However, the countries differ in the implementation of public insurance system, for example the Czech Republic and Slovakia established systems of competitive health insurance funds, Romania introduced one national health insurance fund with district insurance houses, Bulgaria, Hungary, Moldova and Poland operate single national health insurance funds. In all countries, with the exception of Moldova, public health insurance is the dominant source of health care financing.

The countries differ in the level of health care financing measured as a share of total health expenditure on the gross domestic product (Table 2). Romania had the lowest relative health expenditure (5.6% in 2010) in comparison to the other countries in this study that spent on health around 7–9% of their gross domestic product (GDP). Moldova is an outlier, with total health expenditure reaching 11.7% of GDP in 2010. This is the third highest value in the WHO European region. A part of explanation for this could be the low level of GDP and inherited large capacities from the former Soviet style health system. The value of total health expenditure for the WHO European region was 8.3% of GDP and for the European Union was 9.9% of GDP in 2010. On the average, the Eastern European countries spend less on health from their GDP than the Western European countries. That is not surprising as the relation between the total health expenditure and GDP, i.e. an increasing share of total health expenditure on GDP with growing GDP, is well described in the health economics literature.

**Table 2:** Health expenditure and mental health expenditure

**Country**	**Health expenditure as % of GDP in 2010**^**1**^	**Estimates of mental health expenditure as % of health expenditure**^**2**^
Bulgaria	6.9	2.5
Czech Republic	7.9	3.0
Hungary	7.3	8.0
Moldova	11.7	6.5
Poland	7.5	-
Romania	5.6	3.0
Slovakia	8.8	5.0
WHO European Region	8.3	6.3
European Union	9.9	-

Sources: ^1^European Health for All Database; ^2^Jacob et al. [3].

### Universal access

Public health insurance should reduce social and health inequalities through redistribution of financial resources. It seems however, that there are problems in achieving universal access in Bulgaria and Moldova. Bulgaria faces the problem of a large number of non-insured persons that do not pay insurance premium. According to estimates, one million persons are not insured for various reasons [4,5]. Most often, the self-employed persons and the ethnic minorities do not pay premiums. Most of these people receive services through the emergency care. In Moldova, it is estimated that more than 20% of population is not covered by public health insurance. The uninsured include self-employed farmers, occasional employees or unemployed [6]. The minimum package of services is available to the whole population, irrespectively of insurance status. The more generous package under mandatory health insurance is available only for the insured.

### Mental health services

In the WHO European region, the median value of mental health expenditure as a share of total health expenditure was estimated 6.3% [3]. The estimates for countries of Eastern Europe included in this study vary from 2.5% to 8% (Table 2). From this perspective, mental health care is relatively underfinanced in comparison to physical health care in these countries. Moreover, the effect is multiplied: the countries of Eastern Europe spend a lower share of the gross domestic product on total health expenditure and from this amount they spend a lower share of the total health expenditure on mental health services. That is a long-term problem that will not be solved in a short period. Any transfer of money to mental health services is a transfer of money from other health services.

Local experts from all the seven countries report insufficient cooperation between health providers serving to people with mental health problems. An intersectional cooperation is even worse: the social and health care systems work almost independently without clearly defined relations. The frequent problem is a prevalence of biological model instead of social and psychological approach, although the technologies and know-how of modern mental health are known. A poor consensus among the psychiatric profession about the direction of the mental health reforms is a serious obstacle for further development. A poor motivation among the professionals due to low salaries compared with other medical specialties also has to be taken into account [2].

An insufficient level of financing of mental health services leads to absence of financial resources for mental health system development. There were poor investments in mental health services in the past, which led to the situation with a need of renovation of inpatient facilities, and need of improvement of the living conditions in the existing institutions. On the other hand, the national mental health systems also need resources for strengthening weak community mental health services. National mental health systems typically face lack of specialists in child psychiatry and geriatric psychiatry. This is also connected with lack of appropriate residential institutions for these populations.

The available service utilisation data from outpatient services show a great variation among countries. The number of patients contacting outpatient services in Slovakia, the Czech Republic, and Poland per 100 000 inhabitants is much larger than the numbers of patients in Bulgaria and Romania [2]. We also observe a growing number of patients with mental illness served by outpatient services. The countries also differ in the numbers of psychiatric hospital beds (Table 3).

**Table 3:** Psychiatric hospitals beds per 100 000

**Country**	**1985**	**1990**	**1995**	**2000**	**2005**	**2010**
Bulgaria	85.69	89.45	88.96	63.93	67.77	64.88
Czech Republic	140.02	140.02	114.36	113.01	110.39	101.66
Hungary	-	-	98.99	98.57	39.34	32.95
Moldova	137.04	146.27	121.12	74.48	74.77	71.87
Poland	-	-	-	-	67.40	62.56
Romania	98.62	95.36	88.48	81.56	75.46	77.14
Slovakia	-	-	-	93.15	83.54	79.47

Source: European Health for All Database.

### Policy strategies

In Bulgaria, the government adopted *National Mental Health Policy* and *the National Action Plan for Implementation of the Mental Health Policy of the Republic of Bulgaria 2004–2012*. These strategic policy documents built on the *National Mental Health Programme 2001–2004* and the activities stipulated in the First Component of the South-Eastern European Mental Health Project under the Stability Pact [7]. The National Action Plan describes in detail the responsible institutions, ministries and other organizations for all long-, middle- and short-term goals and activities. So far the practice of monitoring the implementation and progress of mental health policy is missing because the accountability to the public and the consumers has not been embraced by either the health administration or the stakeholders yet. The mental health policy by adopting planning and budgeting on a project basis hopes to build the practice of accounting within the principles of governance in the sector.

In the Czech Republic, the Psychiatric Society of the Czech Medical Society formulated the *Concept of Psychiatry* in 2000 [8]. The Concept of Psychiatry was approved by the Scientific Committee of the Ministry of Health in 2002. Deinstitutionalisation and the development of community-based mental health services were the key objectives of the reform. The *National Psychiatric Programme 2007* was announced as a proclamation of the Czech Psychiatric Society in collaboration with the local WHO office. In 2008, the work on the *First Revision of the Concept of Psychiatry* from 2000 was initiated by the Czech Psychiatric Society. The revised version of the Concept of Psychiatry observes that psychiatric care relies mainly on institutionalized services, while community services have not been sufficiently deployed. National mental health policy is missing and, as a result, the development of mental health care is not systematic, psychiatric services are founded with little regard to regional needs, the availability of services is uneven, the mental health is underfinanced and in comparison to some western European countries somewhat delayed [9]. Despite many efforts, no reform document was implemented and the vision of community care was not adopted unanimously by psychiatric profession. In October 2012, the Ministry of Health surprisingly announced a preparation of mental health reform based on the community mental health centres serving population of 100 000 inhabitants. The policy should be implemented in 2014 with help of EU funding.

In Hungary, the first *National Programme of Mental Health* was ratified in 2009 as a Ministry of Health programme, but it has not become an official government programme yet, and has never received any financial support [10]. Community psychiatry, one of the fundamental elements of WHO mental health initiatives, is in its infancy in Hungary, although it is often mentioned in the *National Programme of Mental Health*, but only at a rhetoric level, without any actual plan about how this new paradigm will be introduced and realized [11]. Hungary is in a double-bind dilemma in terms of policy formation. On the one hand Hungary tries to conform to the “European expectations”, on the other hand the existing structures, established operational mechanisms, economic constrains often frustrate the reception of both policies and policy procedures [11]. From the Hungarian Ministry of Health’s perspective, the national mental health policy has to satisfy three criteria: (a) the policy has to have some sort of connection to the WHO mental health initiatives; (b) the policy has to be accepted in the Hungarian mental health community (dominated by hospital psychiatrists); (c) the policy has to be feasible under current economic conditions. The so-called Semmelweis Plan [12], published in 2011, identified paediatric and youth psychiatry as one of the priority areas for the improvement. The Semmelweis Plan states that epidemiology surveys have shown that the average prevalence of mental diseases among children between 4–17 years is 15.8%, i.e. it reaches the level of endemic diseases. At the same time, Hungary does not have an appropriate infrastructure or network of experts to provide efficient care. The overwhelming majority of patients do not have access to appropriate paediatric psychiatric diagnosis and therapy because of the lack and territorial unevenness of care centres.

In Moldova, the *National Program on Mental Health for 2007–2011* was approved by the Government in March 2007. The National Programme on Mental Health [13] aims at decreasing morbidity, mortality and disability rates caused by mental illness as well as improving mental health parameters among population by increasing the accessibility and efficiency of the population to psychiatric care, by integrating people with mental disorders into families and into the community, by raising awareness on mental health issues and by recognizing the mental health issue as being one of the basic interdisciplinary scientific problems. The main objectives of the programme also provide for the reform of the mental health care system, emphasizing deinstitutionalization, non-stigmatization, priority development of outpatient treatment community services, providing differentiated psychiatric care to people with mental health issues, depending on the disease progression stage, offering various treatment opportunities. The National Programme stipulates the existence of financial difficulties in the development of psychiatric care system according to the outlined objectives. Thus, the success of the action plan mainly depends on the adequate funding from health insurance. Nevertheless, it seems that in spite of huge financial problems, poverty, and national debt, government is aware of the importance of health system for the national development and the health budget grows continually.

In Poland, the Mental Health Act was approved in 1994. Until 1994, there was no definitive legal protection for the rights of people with mental health disorders. The *National Programme of Mental Health Protection* was prepared by the Institute of Psychiatry and Neurology and was approved by the Polish Psychiatric Society and the Ministry of Health in 2006. Ideas included in the National Programme of Mental Health Protection give hope to many participants of the mental health system for transformation towards community-based mental health care. A significant reduction of psychiatric hospital beds is planned and consequently daily care units should be created. Daily care is seen as a better way to increase the availability and access to mental health services. According to expert opinions the resources are allocated improperly. In some places, the availability of mental health services is very high while in other areas the access is almost blocked. Great expectations are tied with the idea of the so-called local mental health centres, which are planned to cover a population of 200 000.

In Romania, the mental health reforms have been introduced by the reforms of the whole health system and by the international initiatives and programmes on mental health [14]. The changes introduced by the general health system reform include new ways of financing health services, new payment methods of health providers, reorganization of some long-term care institutions for persons with disabilities, and institutionalization of home-care services. The international mental health initiatives and programmes have been more successful in initiating the development of new models of mental health care in Romania. Still, these developments, initiated by NGOs and financed by external funds, are occasional, focused on specific issues and have insufficient governmental support. An important step for further changes has been the WHO National Mental Health Assessment, which was conducted in 2000. Some recommendations of this assessment have already materialized in the adoption of the *Law on Mental Health Promotion and Protection of Persons with Psychiatric Disorders* and the development of the national mental health strategy. The *Mental Health National Strategy* was developed by the Ministry of Public Health in 2006. The vision is to provide mental health services that are attainable, of high quality and based on the existing needs, and to provide promotion programmes, prevention and education in mental health.

In Slovakia, the *National Programme for Mental Health of the Slovak Republic* was developed and adopted by the Slovak government in 2004 after two years of preparations. The National Programme is a strategic document outlining measures to improve the population’s mental health. The Council for Mental Health was established by the Ministry of Health in 2005. The *Action Plan of the National Programme for Mental Health of the Slovak Republic 2005* was approved by the Slovak government and the Ministry of Health was made responsible for its implementation. The Action Plan outlined 45 general tasks and nine research tasks for the period of the subsequent two years (2006–2007). In 2008, the government evaluated the implementation of the Action Plan and the Ministry of Health prepared an updated version of the Action Plan for the period 2008–2010.

### The role of civic society

In Bulgaria, there is scarce social support practised, mainly inside families, ethnic groups or corporate groups. Although there are some consumer organisations in Bulgaria, the operation of most of which was initiated by health professionals, they have a very limited impact on the quality of care, the allocation of resources and the political decision making at the national or local level [2]. The official documents governing mental health services provision include arrangements providing for the rights of access to services, informed consent, the legal procedures in cases of statutory commitment, access to personal files, etc. However, these rights are not organised in any special documents and the consumers are not informed about having these rights. Data obtained through focus groups indicate that even if the patients have a clear understanding of their rights, for example of their right to look in their personal files, the information there is organised and written up in such a way, that it does not provide any meaningful information for the patients.

In the Czech Republic, there are dozens of patient groups and NGOs with an increasing voice in mental health. For example, Kolumbus is an association of patients and former patients, Sympathea is an association of relatives of the mentally ill, the Czech Association of Mental Health is an organisation of patients, families, and professionals, and FOKUS is a non-profit association of professionals providing community services. NGOs became leaders in establishing community-based services, in providing advocacy and in protecting human rights. Although NGOs do not have large financial resources at their disposal, they contributed to mental health reform in the country. For example the Centre for Mental Health Care Development published its own mental health reform proposal [15].

In Hungary, patients have gained a free choice of a health provider since 1992. In 1997, the Act on Health comprehensively declared the patient rights, for example the right to human dignity, the right to refuse treatment, or the right to get to know the patient file [16]. The institution of patient representatives was also created. Patient groups and associations are getting increasingly active in the country. For example, *Voice of Soul Association*, established in 1996, is a fully user-run and user-controlled NGO that is active in mental health. The *Mental Health Interest Forum* is a coalition of organisations, users and the social sector. Its aims are the advocacy, human rights, trainings and the continuous monitoring of psychiatric institutions and nursery homes. The government involves NGOs more and more in the policy formulation processes, however these involvements are criticized as being formal, serving only for demonstration purposes that users were consulted [11]. Still user groups see themselves as important policy actors that are getting stronger and better at finding strategies for lobbying.

In Moldova, patient groups and advocacy organizations exist in the country; however their role in health policy is rather limited. The health sector in Moldova benefits from a range of international donors. The UN agencies, such as UNICEF, UNFPA, World Health Organisation and European Union (TACIS programme) provide both material and technical support. Major financial support is also provided to health related activities bilaterally from governments such as the US and Japan. The World Bank provides support to the health sector through large scale loans for health reform activities in the health sector. International NGOs such as the Red Cross, Pharmacists without Borders and the Soros Foundation have also made important contributions to health assistance [6].

In Poland, the number of NGOs and the scope of their activities have been growing since the 1990s. NGOs lead many initiatives in creating community-based services, in providing advocacy and in protecting human rights of people with mental illness. Although NGOs do not have large financial resources, they contributed the most to mental health reform in the country. Examples of NGOs active role in mental health are the Coalition for Mental Health, Faith and Light, Saint Albert or Curatus. Some patients groups are financed by the industry, therefore their initiatives can be rather aggressive and they may lobby on different issues [2].

In Romania, mental health system fails to protect human rights and dignity of people with mental illness. This has often been highlighted by many NGOs and by Amnesty International. Living conditions in psychiatric wards and hospitals were mentioned by the EU progress report on Romania’s accession. In response to these shortcomings, the Ministry of Public Health intends to upgrade some of the inpatient and long-term care facilities in parallel with the development of community-based care. However, despite the human rights obligations and policy commitments of the government, the enjoyment of the right to mental health care remains more of an aspiration than a reality [14]. The main challenges are: tackling the stigma and discrimination associated with mental illness; bringing some coherence to the system of mental health care where it extends across more than one government department: ensuring that, in parallel with the development of community-based mental health services, the state benefit system evolves to support new emerging models of service provision; ensuring that the justice system is able to fulfil its prescribed role in the administration and monitoring of compulsory detention and treatment under mental health legislation [14].

In Slovakia, there are several patient groups with an increasing voice in policy-making, service provision, and advocacy. Examples include PREMENY, an association of mental health care users and providers, OPORA, an association of families and friends of people with mental illness, ODOS – Open the Doors, Open Your Heart, an organisation implementing an anti-stigma programme, and the League for Mental Health that serves as an umbrella organisation of more than 30 NGOs active in mental health, DRUHY BREH, a bi-monthly journal for patients, relatives and mental health professionals [17]. These initiatives are able to bring more attention to mental health, but are less likely to be able to influence allocation of resources. Some NGOs take part in the Council for Mental Health (an advisory body of Minister of Health) that should implement the National Programme for Mental Health of the Slovak Republic.

## Discussion

### Mental health policy

The social and economic transition in the 1990s initiated the process of new mental health policy formulation and adoption of mental health legislation stressing human rights of patients (Table 4). In all countries, we observe a strong call for “a pragmatic balance” of community and hospital services [18,19]. We can observe that the know-how about modern mental health care and about direction of needed reforms is available in documents, policies and programmes. However, this does not mean real implementation. Two positive facts support the reforms: firstly, the existence of small but motivated groups of professionals aiming to continue with a change of the mental health system, and secondly, international support from the European Union, WHO and large international NGOs. Bulgarian and Romanian mental health policies have benefited from the EU membership, because mental health has been an important issue during the accession process. A strong criticism against the poor conditions in the Bulgarian and Romanian mental health institutions was expressed by a number of international NGOs, media and EU representatives. This has led to the improvement of human rights for the mentally ill and the introduction of mental health reforms with international help.

**Table 4:** Health policy documents and legislation

**Country**	**Strategic policy documents**	**Patient rights legislation**
Bulgaria	National Mental Health Programme 2001, Mental Health Policy 2004	Health Care Act 2004
Czech Republic	Concept of Psychiatry 2000 (Revised 2008), National Psychiatric Programme 2007	General Health Care Act (amended several times)
Hungary	National Mental Health Programme 2007	Health Care Act 1992, 1997
Moldova	National Mental Health Programme 2007	Mental Health Act 2008
Poland	National Mental Health Programme 2006	Mental Health Act 1994
Romania	Mental Health Strategy and Mental Health Action Plan 2006	Mental Health Act 2002, 2006
Slovakia	National Mental Health Programme 2004, Mental Health Action Plan 2005 and 2008	General Health Care Act (amended several times)

### Mental health services financing

Mental health services are financed from the public health insurance in the Czech Republic, Hungary, Moldova, Romania, Poland, and Slovakia as any other health services. There is no separate budget for mental health. Bulgaria is the country with dual financing of mental health services. The inpatient care for the mentally ill is financed from the budget by the Ministry of Health and the outpatient care is financed from the public health insurance. The centralised state hierarchy of national health services was replaced by a new bureaucracy of public health insurance in all seven countries. Public health insurance is primarily oriented to reimbursement of health services according to detailed lists of services. However, the bureaucracy of health insurance and of social system can curb the development of new initiatives and approaches that are not in line with the existing administrative and financial division between health and social sectors and with the existing administrative and financing procedures. This argument is in particular important for mental health where a transitional change from institution-based to community-based care is needed and a link between health and social sectors is essential [7].

### Civic society

The general acts on health or specific acts on mental health declared the patient rights (Table 4). Patients gained, for example, a free choice of health care provider or access to personal files. The institution of patient representatives was established in various forms. In contrast to the development in the Western Europe, the civic society was suppressed and NGOs and similar organizations were practically non-existent or under governmental control in the communist society in the 1980s. In the present, there are dozens of NGOs with increasing voice in mental health policy and health services provision. However, despite many positive developments, some patient rights remain more of an aspiration than a reality.

## Conclusions

The countries of Eastern Europe included in the study have gone through social and economic transition in the last two decades. The countries share their common totalitarian history that caused health, social, economic, and moral deterioration. The burden of totalitarian history still influences many areas of social and economic life, which also has to be taken into account in mental health policy. In contrast to the development in the Western Europe, the civic society was suppressed and NGOs and similar organizations were practically non-existent or under governmental control. One important observation is that the totalitarian past has a longer future than it was initially expected [17,19,20]. Observing the differences between the countries, we have to agree with the view that the assumed similarity between former communist countries was more fictional than real, resulting from a suppression of differences. What appeared to be similar was, in fact, a misleading impression created by biased data, obstructed access to information and the manipulation of findings [19].

There is no doubt that the global economic crisis has had a significant impact. Although economic crisis is not a positive event that is welcomed by societies, an economic crisis can be seen as an opportunity for new necessary system reforms in the health care, including mental health care. The countries face many problems and obstacles in reforming their mental health services. Some of the countries had some initial advantages, some of them started the reforms earlier, but it does not at all mean that the mental health reform will be more successful. The social and economic transition in the 1990s initiated the process of new mental health policy formulation and adoption of mental health legislation stressing human rights of patients. However, not all of this has been successfully realised and we may observe that after twenty years of health reforms and reforms of health reforms, the transition of the mental health systems still continues. In spite of many reform efforts in the past, a balance of community and hospital mental health services has not been achieved in these countries yet.

## Competing interests

The author declares that he has no competing interests.

## Pre-publication history

The pre-publication history for this paper can be accessed here:

http://www.biomedcentral.com/1472-6963/14/42/prepub
